# Occupational therapy in pain management: An exploration and description of current UK practice

**DOI:** 10.1177/03080226241300837

**Published:** 2024-12-03

**Authors:** Rebecca Slee, Alison Warren, Miriam Noonan, Tristan Henderson

**Affiliations:** University of Plymouth, Plymouth, UK

**Keywords:** Pain management, chronic pain, occupational therapy, practice, online survey, United Kingdom

## Abstract

**Introduction:**

There is a growing body of evidence regarding the contribution to the management of chronic pain by occupational therapists. However, there is limited research available regarding contemporary practice in the United Kingdom (UK).

**Method:**

An on-line questionnaire was circulated via social media, profession specific publications and specialist interest groups. Responses were requested from UK-based Health and Care Professions Council registered occupational therapists who identified working with those living with chronic pain and their friends, family and supporters.

**Findings:**

A total of 26 occupational therapists responded. Occupational therapists are working in diverse service settings, offering a range of interventions and recognised the unique contribution by occupational therapists to this area of practice. Perceived barriers to delivering intervention included staffing levels, understanding of the occupational therapists’ role and suggestions for additional interventions were stated.

**Conclusion:**

This research increases understanding of UK-based occupational therapist’s current practice in pain management and views of their role. It provides considerations for further research including how to increase awareness of occupational therapy’s contribution to this area of practice, exploring the occupational needs of individuals and those of friends, family and supporters to ensure intervention is designed to best meet the needs of this population.

## Literature review

Chronic pain remains a global health priority, affecting an estimated 30% of the global population (The Lancet, 2021). A recent United Kingdom (UK) study conducted by the Independent Polling System of Society on behalf of the British Broadcasting Corporation (BBC) suggests that the figure is closer to a quarter of the population in the UK (BBC News, 2022). Therefore, it is imperative to provide effective and accessible treatment for this population. The World Health Organization’s updated definition of chronic pain in the 11th edition of the International Classification of Diseases-11 provides classification of chronic pain as an overarching diagnostic code, with seven sub-codes (Treede et al., 2019). Previous diagnostic codes for chronic pain were considered to not reflect its epidemiology; therefore, adversely impacting accurate data collection, hindering the development and implementation of new therapies (Treede et al., 2015). The new classification has the potential to improve diagnosis of chronic pain conditions internationally (Barke et al., 2022), thus informing service pathways, increasing accessibility to support and treatment for those persons living with chronic pain.

In theUnited Kingdom, the National Institute of Health and Social Care Excellence (NICE) developed guidance on assessment and treatment of chronic primary and secondary pain, acknowledging these conditions can co-exist (National Institute for Health and Care Excellence, 2021). NICE recommendations include a person-centred assessment, development of a care support plan and discussing how pain affects life and how life affects pain (National Institute for Health and Care Excellence, 2021). The UK Faculty of Pain Medicine (FPM) (Faculty of Pain Medicine, 2021) identifies the importance of including occupational therapists in the multi-disciplinary pain management team, recognising the impact chronic pain may have on a persons’ ability to engage in meaningful occupations. This is supported by a growing international evidence base regarding the contribution that occupational therapists make within pain management teams (Hill, 2016; Lagueux et al., 2018). A recent study by Lagueux et al. (2023) described current occupational therapy practice in Canada, concluding that occupational therapists working in this area were encouraged to continue to increase the evidence base and advocate for recognition of their role in supporting those living with chronic pain.

Within the United Kingdom, Goodall and Brown (2022) discuss the benefits participants perceived in attending occupational therapy-led sessions that were part of a pain management programme. However, Jain et al. (2022) identify inequalities and inequities in chronic pain services available in areas of deprivation within the United Kingdom, acknowledging that occupational therapists are not always part of pain management teams.

This research aimed to present contemporary UK occupational therapy practice, as well as explore occupational therapists’ perceptions of their current role and consider which interventions they may wish to offer in future.

## Method

A cross-sectional survey was developed with advice and direction provided by a stakeholder group, and this included those with lived experience of chronic pain and Health and Care Professions Council (HCPC) registered occupational therapists working in this area of practice. Closed and open-ended questions were used to elicit demographic information of survey respondents, areas of practice, referral routes, service provision and types of intervention. Free text sections sought to understand if occupational therapists perceive any limitations or barriers in the support they offer to people living with chronic pain and their friends/family supporters.

The survey was piloted and amendments were made to wording of questions and functionality of the Joint Information Systems Committee (Jisc) software. The survey took approximately 10–15 minutes to complete and was suggested to optimise engagement (Toepoel, 2015: 36).

## Recruitment and participants

Following the University of Plymouth’s protocol, the survey was distributed online via social media posts, the Royal College of Occupational Therapists (RCOT) specialist section e-newsletter, the RCOT *OTnews* publication and networking forums including research groups and the specialist section occupational therapy pain forum group.

## Ethics

Ethical approval was granted by the University of Plymouth Faculty of Health Staff Research Ethics and Integrity Committee (3545). Details of the study including the purpose, data protection and storage were provided to all participants at the start of the on-line survey and informed consent was obtained prior to respondents submitting the survey.

## Data analysis

Anonymised survey responses were exported to an Excel password protected University of Plymouth Onedrive. Descriptive statistics were produced from closed questions. Coding was used to produce themes from the data generated by the qualitative questions (Jacobsen, 2017). These methods of description and analysis were used to provide insight into occupational therapists’ current and future practice in pain management in the United Kingdom.

## Results

### Demographics, professional education and training

A total of 26 occupational therapists responded to the survey. The majority of respondents were female *n* = 23, male *n* = 2, there was one omitted response. Most occupational therapists qualified within the United Kingdom (*n* = 24), 85% of respondents identified their ethnic group as white British and 15% as white. A total of 52% (*n* = 14) of respondents had over 20 years’ experience in practice, 32% (*n* = 8) over 10 years, 8% (*n* = 2) 7–8 years, the remaining respondents had a minimum of 3 years’ experience.

Respondents worked in a diverse range of practice settings (see Table 1).

**Table 1.** Occupational therapists demographics, practice settings and training.

Gender	*n*	%
Female	23	92
Male	2	8
Ethnic group		
White British	22	85
White	4	15
Trained in the UK	24	92
Years of working as an occupational therapist since graduating		
20 years + experience	14	52
10 years+	8	32
7–8 years	2	8
3–6 years	2	8
Service settings		
Specialist pain service	13	46
Rheumatology	4	14
Community mental health	2	7
Social care	1	4
Urgent community response	1	4
Private practice	1	4
Other specified as Specialist adult and children’s Chronic Fatigue Service, community pain management, Chronic Fatigue outpatient service, further education, outpatient hand therapy, mental health liaison	6	21
Pain management education as part of occupational therapy course programme		
No	20	77
Yes	6	23
Training undertaken as CPD post registration		
Yes	21	81
No	5	19

CPD, continuous professional development.

Most occupational therapists’ pre-registration programmes did not include education on working with those living with chronic pain as part of their occupational therapy programme (*n* = 20). Most respondents reported specialist training was undertaken post registration as part of Continuous Professional Development (*n* = 21). Training varied from shadowing within specialist services to more formalised routes of attending special interest group study days, post-graduate study and educational webinars.

### Referral routes to services

Respondents were asked to identify the most frequent source of referrals to the service they work in, they were able to select more than one response. A total of 36% (*n* = 19) of respondents received referrals from general practitioners, 26% (*n* = 14) from other health care professionals including physiotherapist and psychologists or pain consultants (see Table 2). Only two respondents specified they received self-referrals.

**Table 2.** Referral routes to services and average weekly referral numbers.

Referral routes to services	*n*	%
General practitioners	19	36
Other health professionals including physiotherapist, psychologists	14	26
Pain consultants	10	19
Social care professional	2	4
Other specified as pastoral support, accident and emergency and case manager, pain nurses and other consultants	6	11
Self-referral	2	4
Average number of referrals to services per week		
6–10	8	32
0–5	6	24
Over 30	4	16
21–30	3	12
11–15	3	12
16–20	1	4
What percentage of these were referred to occupational therapy		
100	15	60
67%	1	4
50%	2	8
33%	3	12
17%	4	16

The highest number of responses suggests services received between 6 and 10 referrals a week (*n* = 8; see Table 2). However, there is a spread of responses in other categories, indicating variation of between 0 and 5 (*n* = 6) to >30 (*n* = 4) referrals per week. Sixty percent of respondents (*n* = 15) reported that referrals were triaged or directly referred to occupational therapy.

Most respondents are the first professionals whom those living with chronic pain will meet in their service pathway (92% (*n* = 23)). Respondents identified using occupational therapy-specific assessments in their initial assessments 35% (*n* = 8). The most frequently reported were in-house occupational therapy designed assessment 40% (*n* = 4), the Canadian Occupational Performance Measure (COPM) (Law et al., 2019) and an assessment designed based upon the Model of Human Occupation (Kielhofner, 2008) also used (see Table 3).

**Table 3.** Practice overview of UK occupational therapists.

Descriptions of practice	No of responses	Percentages
Initial assessments		
Any named professional generic assessment	8	35
Biopsychosocial assessment	3	13
Occupational therapy assessment	8	35
Other standardised self-administered questionnaire	4	17
Occupational therapy assessments		
Occupational therapy in-house designed initial assessment	4	40
Canadian occupational performance measure*	3	30
Model of human occupation informed assessment**	3	30
Do you routinely identify who are friends/family supporters?		
Yes	24	92
No	2	8
Who do you involve in occupational therapy intervention?		
Individual and their friends/family supporters	19	73
Individual	7	27
The friend/family supporter	0	
Types of intervention		
Pacing advice	26	10
Sleep hygiene	24	10
Education about pain mechanisms	23	9
Request for reasonable adjustments at work	20	8
Signposting to community services	20	8
Referral to support for mental health	20	8
Group pain management programme	16	6
Vocational rehabilitation	16	6
Advice on benefits	16	6
Environmental adaptation	14	6
Equipment provision	14	6
Completion of fit note or allied health professional health and work report	11	5
Group exercise programme	4	2
Introduction to mindfulness techniques	2	1
Goal setting	6	2
Grading exercise/activities	4	2
Referral to social care	2	1
Fatigue management	2	1
Relaxation techniques	3	1
Other	5	2
Types of intervention offered to friends/family supporters		
Any the patient wishes	5	19
Individual sessions with the patient/service user	12	46
Group sessions with service user	5	19
Assessments	3	12
Carer support	1	4

* Law et al. (2019).

** Kielhofner (2008).

A number of respondents routinely identify who the friends, family and supporters are of those living with chronic pain as 92% (*n* = 24) and include friend, family and supporters in their interventions as 73% (*n* = 19), with 27% (*n* = 7) offering intervention to those living with chronic pain solely. Respondents reported that service users were offered both individual and group sessions. The number of sessions offered varied from 1 to a maximum of 17. A range of interventions were offered to those accessing services with pacing advice, sleep hygiene and education about pain mechanisms the most frequently reported (see Table 3). There was also a range of interventions that friends, family and supporters could engage in as agreed by the service user or as part of a group programme; however, only one respondent reported offering specific intervention for carer support.

### Scope for other interventions

**Figure 1.**
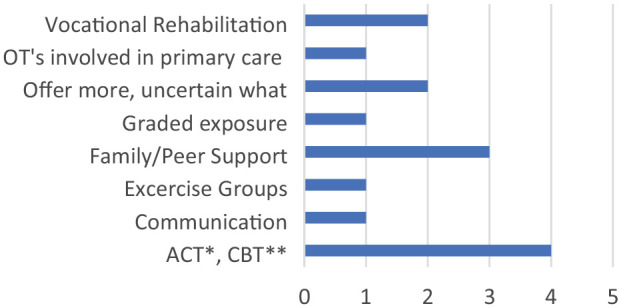
Interventions offered by occupational therapists. *Acceptance and commitment therapy. **Cognitive behavioural therapy.

A number of respondents, about 70% (*n* = 14) stated they would like to offer additional interventions, and there was variation in the types of interventions suggested (see Figure 1). Two respondents were uncertain about what they could offer, stating this was due to lack of knowledge about interventions provided in other services.

The questionnaire included free-text options to consider occupational therapists views on their practice and what, if any potential barriers there are to offering intervention. Occupational therapists’ unique skill set in being one of the few/only professions who are dual trained was highlighted in the qualitative responses. Respondents explained that occupational therapist’s professional education in both physical and mental health was particularly relevant in pain management.

Our training and background in both mental and physical health puts us in the perfect position to deliver a truly biopsychosocial approach. Respondent 109123362

Truly person centred, dual training of OTs is imperative to understanding the biopsychosocial impact of pain on individuals. Respondent 109123834

As a result of this biopsychosocial approach, respondents perceived they were well positioned to consider the needs of both the individual and their friends, family and supporters. Respondents discussed that occupational therapists are holistic in their assessment and practice. Thus, considering all aspects of some-one’s life, what is meaningful to them and that of their friends, family and supporters.

I think that we look at the whole person, impact of pain on function, look at goals based on meaningful activity and consider impact on wider family. Respondent 112080786

Holistic, involves all areas of patients life, works from needed or desired activity up to function. Respondent 107123074

Occupational therapists are trained in activity analysis, meaning they can facilitate engagement in meaningful occupation at the right level to meet a person’s needs. Responses in this questionnaire aligned with this as respondents reported that their intervention in pain management was focused on understanding and enabling meaningful occupation. They perceive their unique contribution is providing intervention that supports re-engagement/adapted engagement in valued activities.

It’s not about symptom management it’s about living my life and doing the things that matter to me . . . that is what is going to keep someone well. Respondent 112044124

Insight into impact on occupations and analysing how to perform occupations while dealing with chronic pain. Respondent 109838508

Respondents were asked what/if any are the barriers to offering occupational therapy within this area of practice. Respondents reported staff shortages, not always being part of multi-disciplinary teams in pain management services, limited understanding of the role of occupational therapy and service users ability/readiness to engage with occupational therapy were all barriers to engagement in occupational therapy intervention.

Respondents reported that the role occupational therapy has in pain management may not be as widely understood. The scope of practice may be unclear within multi-disciplinary teams but also to potential service users. Respondents attributed this to:

Others understanding of Occupational Therapy as a profession as well as the lack of OTs working within this area. Respondent 109123362

Dismissiveness in attitudes of other professionals sometimes!. Respondent 109891104

Feeling prepared to discuss treatment options, therapeutic relationship. Respondent 109820393

Patient sometimes resistant to idea of mind/body link and their efficacy in self managing. Respondent 109814935

## Discussion

The findings present the current practice of 26 UK-based HCPC registered occupational therapists in the area of chronic pain. The demographic data collected about the respondent’s demonstrated homogeneity with the majority of respondents identifying as white and female which aligns with current demographics within occupational therapy in the UK (HCPC, 2021). The lack of diversity within the profession has been acknowledged as a priority that must be addressed, aiming to reduce inequality and increase inclusivity, both within the profession and to better represent the population occupational therapists serve (Atwal et al., 2021). Backstrom et al. (2023) discuss that gender disparity within the occupational therapy profession was not the most pressing issue for participants in their research, but broadening the scope of the profession to create a more diverse professional culture was important now and in the future. The RCOT recognises the need to increase diversity in its UK workforce and in response has developed an Equality, Diversity and Belonging strategy (Royal College of Occupational Therapy, 2023a). However, this does not guarantee that the occupational therapy workforce and therefore potential research participants would be more representative of the general population.

Occupational therapists discussed that their pre-registration education enabled a biopsychosocial approach in supporting those living with chronic pain. The British Pain Society’s standards for Pain Management Programmes identifies how occupational therapists’ professional qualification indicates they are ideally placed to work in this area of practice (British Pain Society, 2013). Furthermore, Health Education England’s roadmap to practice for occupational therapists working in First Contact Practitioner roles sets out the development of core clinical skills for assessment and treatment of chronic pain in older adults (Health Education England, 2021), indicating this is an expanding area for occupational therapy roles.

Whilst the unique ability of occupational therapists to be holistic due to their dual training was recognised, many respondents within this study reported they needed to undertake further continuous professional development to increase their understanding of chronic pain. Although explaining pain mechanisms is not specifically an occupational therapy domain of practice, it is considered important in collaborative practice to support those living with pain (Kang et al., 2023). Another explanation identified by this research is that professional pre-registration education programmes do not include learning opportunities specific to chronic pain and pain management, but it may be considered when examining case studies, specialist areas of practice or within interprofessional modules. However, the need to enhance clinical skills for those working in this area is endorsed by the FPM’s recommendation for occupational therapists to develop appropriate areas of knowledge as set out by the International Association for the Study of Pain’s core curriculum for occupational therapists (Faculty of Pain Medicine, 2021). Thus creating a need for occupational therapist to undertake education and training specific to chronic pain.

Respondents of this research work in diverse practice areas within the United Kingdom, which may be representative of the prevalence of chronic pain within the general population. Another consideration is that chronic pain is likely to impact an individual’s occupations; therefore, they are more likely to be referred to occupational therapists. Occupational therapists working in mental health settings were represented, which aligns with the Societal Impact of Pain joint statement on Pain and Mental Health (Societal Impact of Pain, 2023), this calls upon national and European Union policy makers to include assessment of chronic pain within mental health services and to integrate services. There was significant variance in the number of referrals to services overall, potentially due to the differing service settings occupational therapists work in, indicating heterogeneity in referral pathways. As suggested by the responses, a consideration may also be the level of awareness of occupational therapy’s scope of practice among other health professionals and service users. As discussed by Hill (2016), further understanding of the role and scope of occupational therapy practice in this field is needed. Within the UK strategy for Allied Health Professions (National Health Service, 2022) professions are being called upon to articulate their offering to individuals, communities and other health and social care professionals through accessible means. As these populations are likely to utilise differing means of accessing information, further research is needed to consider preferences and what might be most accessible way of amplifying the voice of occupational therapy in pain management. Ensuring third sector support services are aware of what intervention is available is one potential area for consideration, another could be encouraging individuals to share their stories about the value of occupational therapy intervention across their networks, including social media platforms.

Lagueux et al. (2023) suggest that occupational therapist’s practising in Canada have an improved understanding of their distinct role in Pain Management, within this research occupational therapists identified their contribution to this area of practice. However, they acknowledged their role was not as valued as other professions. One explanation for this may be related to the under-representation of occupational therapists within pain management services, a phenomenon discussed by Jain et al. (2022). An alternative consideration is that referral routes lack clarity and a lack of knowledge both inter-professionally and of service users of the role of occupational therapy in pain management is potentially a factor inhibiting accessibility as indicated by occupational therapists in this study. Raising the awareness of the role of occupational therapy within interprofessional teams can be enhanced through service audits as part of quality improvement and outlining specifically where occupational therapy intervention is key to supporting individuals accessing services.

A range of assessments are conducted by occupational therapists. The most frequently reported occupational therapy standardised assessment used being the COPM (Law et al., 2019), a tool advocated as a means of measuring occupational participation and engagement in those experiencing chronic pain (Lagueux et al., 2018).

Within this research occupational therapy, non-standardised initial assessments were also used, although the research did not capture information about the distinct features of these assessments. This finding provides a potential area for further research regarding the distinct features of non-standardised assessments, exploring what unique attributes they offer occupational therapists as they strive to understand from the individual’s perspective the impact that chronic pain has on occupational performance and occupational identity.

Thompson et al. (2020) discuss recognising occupations aligned with values linked to an individual’s self-identity has the potential to improve the quality of life of those living with chronic pain. As acknowledged in the qualitative data of this study occupational therapists support individuals to consider what matters to them and how maintaining or engaging in these occupations are central to ‘living well’. This aligns with other research that occupational therapy sessions as part of a group programme that focused on engaging in activity and applying activity management were perceived to be beneficial (Goodall and Brown, 2022). Findings from a Lifestyle Redesign occupational therapy intervention indicated an improvement in the quality of life and perceived ability to engage in activity despite pain (Uyeshiro Simon and Collins, 2017). Thus, suggesting that occupational therapists have a role to play in the development/reframing of an individual’s self-identity. The focus on meaningful occupation as a means of promoting health and well-being is a central concern of occupational therapists in this area of practice, as is advocated for the profession. (Royal College of Occupational Therapy, 2023b).

Occupational therapists address social factors of the individual including friends, family and supporters in the initial assessment and interventions. Consideration of connecting and contributing to social relationships that inform occupational choices of culturally diverse people is a central concern of occupational theory and practice (Whalley Hammell, 2014). However, in the majority of practice settings, this is decided by the service user as opposed to the service user and supporter. Only one respondent specified providing carer-specific interventions, although friends and family sessions were included in the group programmes. Smith et al. (2021) suggest further research is needed into the support available to the friends, family and supporters of those experiencing chronic pain. Social support can potentially be a protective factor in the continuation of self-management strategies post-discharge from services (McMurtry et al., 2020). Another important consideration in chronic pain, due to its pervasive nature, is that self-management approaches may need to be adopted across someone’s lifespan particularly when an increase in the pain is experienced. Therefore, those in supporting roles could potentially encourage re-engaging with what has been previously adopted by the individuals as their self-management approach. This research suggests that those living with chronic pain are opting to include their supporters within intervention; however, only one respondent identified they provided carer-specific intervention. This indicates that further research is needed to explore why this might be the case and to consider the occupational needs of both those living with chronic and their supporters. A further area for research could consider whether this finding was unique to occupational therapy or whether other professions provide interventions for supporters. The voices of service users and those that support them is essential to understand their support needs, thus fully appreciate the efficacy of occupational therapy and identify areas for development of interventions.

Central to occupational therapy practice is the dynamic interaction between the therapist and the service user. Rapport and the therapeutic relationship are key to enable collaborative and client-centred practice (Taylor, 2020), occupational therapists use these skills to establish an individual’s level of engagement. Within pain management for those seeking a more biological medical model intervention in their pain management plan, occupational therapy might not be the initial right approach. As an individualised care plan is recommended in pain management (National Institute for Health and Care Excellence, 2021), this must be formulated collaboratively with service users. Therefore, those living with chronic pain need an understanding of all professionals’ distinct role and how to access support to enable informed choices to be made regarding their self-management (Faculty of Pain Medicine, 2021). The development of Allied Health Professional leadership roles creates potential to increase understanding throughout services of the value of occupational therapy both within interdisciplinary teams and service users. However, UK occupational therapists must continue to amplify their voice in this area of practice to increase access and reduce inequality as occupational therapist’s unique contribution to those living with chronic pain and their friends, family and supporters is further recognised.

### Limitations

The data generated by this research has described some aspects of UK occupational therapy practice; however, there are limitations. The response rate to the survey was low which may contribute to the homogeneity of the sample and therefore results are not generalisable; however, this does appear to be reflected in the percentage response rates of other similar studies (Lagueaux et al., 2023; Mackenzie, 2021). Occupational therapists may be working with those living with chronic pain, which is a symptom of a long-term health condition, as the health condition is the primary problem they may have chosen not to respond to this survey.

The survey was available online, and this may not have been inaccessible to some potential participants. Consideration was given to this method of data collection, and as most UK households have access to the internet (Ofcom, 2021), this was chosen over a mail/paper-based survey. It was also chosen to widen participation across the United Kingdom and reduce time/cost to participants.

The questions were not mandatory; therefore, some questions were not answered by all participants. This created variation in response rates across the dataset, percentage responses were analysed as a complete dataset for each question.

## Conclusion

This survey highlights the current breadth of UK occupational therapy practice settings, assessments and interventions. It demonstrates that occupational therapists perceive their education supports them, providing underpinning theory and understanding aligned to this area of practice. It highlights some of the barriers experienced by occupational therapists and considers areas of future research. These include incorporation of learning opportunities specific to chronic pain/pain management in occupational therapists’ pre-registration education programmes and increasing the occupational therapy scope of practice to include development of interventions to support friends, family and supporters. It also highlights that occupational therapists should continue to amplify their voice, demonstrating their value and contribution within this area of practice.

**Key findings**

- UK occupational therapists in chronic pain management work in diverse practice settings and offer a breadth of generic and specialist interventions both within individual and group sessions.
- Occupational therapists believe they have an important and unique role in pain management teams due to their holistic/dual training and underpinning biopsychosocial approach when working with individuals living with chronic pain.
- Potential areas of further research include pre- and post-registration education may be needed due to the prevalence of chronic pain in the population. Exploration of the occupational needs of those living with chronic pain and their friends, family and supporters.

**What has the study added**

This study has described aspects of current HCPC registered UK-based occupational therapy practice supporting those living with chronic pain and their friends, family and supporters. It has highlighted strengths within practice and potential areas of future research.
